# T-Cell Prolymphocytic Leukemia: An Overview of Current and Future Approaches

**DOI:** 10.7759/cureus.13237

**Published:** 2021-02-09

**Authors:** Ana Colon Ramos, Kidist Tarekegn, Amandeep Aujla, Katherine Garcia de de Jesus, Sachin Gupta

**Affiliations:** 1 Internal Medicine, St Barnabas Hospital, The Bronx, USA; 2 Medical Oncology and Hematology, Hartford Healthcare - Backus Hospital, Norwich, USA; 3 Hematology and Oncology, NYU Langone Health, Long Island, USA; 4 Hospital Medicine, Tower Health Reading Hospital, West Reading, USA

**Keywords:** t-cell prolymphocytic leukemia, alemtuzumab, cytogenetic profile, refractory, relapsed, jak-stat, bcl2, hdac inhibitor, tcl1, mtcp1

## Abstract

T-cell prolymphocytic leukemia (T-PLL) is a rare mature T-cell hematologic neoplasm with a very poor prognosis and limited treatment options to date. Single-agent alemtuzumab remains the first line of therapy for the treatment-naive and relapsed/refractory patients. Prospective clinical trials are difficult to conduct given that these patients have a short life expectancy after the initial diagnosis. As a result, researchers are implementing the use of targeted therapies in vitro and ex vivo followed by in vivo trials on a small subset of patients which are reviewed here. Newer approaches in the treatment of T-PLL are developing based on recognizing the cytogenetic phenotype of each patient and targeting the identified defective genes that are usually involved in the cell cycle regulation such as protooncogenes, tumor suppressors, and deoxyribonucleic acid (DNA) repair genes. These could potentially redirect the management in the near future and improve the overall survival (OS) and the progression-free survival (PFS) for these patients.

## Introduction and background

T cell prolymphocytic leukemia (T-PLL) is a very rare, mature T cell leukemia of small to medium size lymphocytes that shows post-thymic features. It involves the peripheral blood, lymph nodes, spleen, liver, skin, pleura, central nervous system (CNS), and bone marrow. T-PLL cells express a post-thymic (TdT-, CD1a-), mature T-cell immunophenotype (CD2+, CD5+, CD7+, CD16-, and CD56-). The leukemic lymphocytes are predominantly CD4+/CD8-. However, there are lymphocytes that are CD4+/CD8+ or CD8+/CD4-. In addition, in very rare cases, we can find CD4-/CD8- phenotype [1]. T-PLL cases are negative for the T-cell Intracellular Antigen 1 (TIA-1) and the human T-cell leukemia virus type 1.

Splenectomy, splenic irradiation, leukapheresis, and alkylating agents such as the included in the CHOP (cyclophosphamide, doxorubicin, vincristine, prednisone) regimen have been used in the past for the treatment of T-PLL but were found to be ineffective. Later on, purine analogs such as fludarabine, cladribine, and pentostatin were introduced for the therapy of these disorders showing slightly improved but still limited response. Finally, alemtuzumab, a monoclonal antibody, was found to achieve complete remission in the majority of the patients but still does not represent the cure for this disease [2].

T-PLL has a very low incidence and poor prognosis with patients not often living for a very long period of time after the initial diagnosis. Furthermore, prospective study trials are difficult to conduct, therefore the currently available treatment options are limited. It is important for the patients and for the field of hematology to discover and better define the therapeutic options that can be used in these patients. Recently, researchers are implementing the use of targeted therapies in vitro and ex vivo followed by in vivo trials on a small subset of patients [3]. These new treatment options are based on the cytogenetic profile recurrently expressed and have shown promising results that may lead to reorganization of the existing protocol for the diagnosis and treatment of T-PLL in the near future.

Pathophysiology

The pathophysiology of this disease is not well understood. The most common cytogenetic features in T-PLL are recurrent changes involving chromosome 14. These include inv(14) (q11q32) and t(14;14) (q11;q32). These alterations lead to the activation of the proto-oncogene TCL-1 [1] which plays an important role in the pathogenesis of T-PLL. Its overexpression results in the promotion of cell proliferation through activation of the protein kinase B (Akt). Moreover, it serves as a cofactor of Akt1, enhances Akt1 kinase activity, and promotes its nuclear transport. Genomic alterations of the tumor suppressor ataxia telangiectasia mutated (ATM) were also found in >85% of cases. Other important genetic alterations include activating mutations targeting the Janus kinase (JAK)/signal transducer and activator of transcription (STAT) pathway [4]. All these mutations suggest the pathogenetic mechanism by which T-PLL is developed.

Clinical features

T-PLL is estimated to account for around 2% of the mature lymphocytic leukemias commonly seen in older adults with a slight male predominance [5]. The median age at presentation is estimated to be 65 years. 

Typically T-PLL presents with rapidly increasing leukocytosis ( typically >100,000/ul), anemia, thrombocytopenia, hepatomegaly, splenomegaly, and generalized lymphadenopathy. Moreover, extranodal presentations include skin infiltration, pleural/peritoneal effusions, central nervous system (CNS) involvement, peripheral and periorbital edema. There is a smaller proportion of patients who have an initial indolent course [6], [7].

Current standards of treatment

T-PLL cannot be cured with current widely used treatment options. For this reason, treatment is reserved for patients with symptomatic or active disease. In addition, early treatment of asymptomatic disease has not been shown to improve clinical outcomes [8].

Indolent Disease

These patients inevitably progress within one or two years, sometimes with a rapid course that may be fatal. For this reason, close monitoring is recommended with complete blood count at monthly intervals along with the clinical examination [8].

Management of Active Disease

Without treatment, patients with active T-PLL progress quickly with a median overall survival (OS) measured in months. Therapy is offered to alleviate the symptoms and improve OS. 

Alemtuzumab: Initially known as CAMPATH-1H, first approved as a medication for B-cell chronic lymphocytic leukemia in 2001 [9] is a humanized, unconjugated, IgG1 monoclonal antibody against surface antigen CD52, a nonmodulating antigen present on B and T lymphocytes, the majority of monocytes, macrophages, natural killer (NK) cells but not in hematopoietic progenitor cells. It can induce cell death in vitro by activation of the classical pathway of the complement system, opsonization with phagocytosis, and antibody-mediated cell toxicity [10]. Used as a single agent intravenous therapy the overall response rate (ORR) will be 90% with complete remission (CR) in the majority of patients [11]. Thirty-nine patients from a number of European countries with T-PLL who received CAMPATH-1H treatment between March 1993 and May 2000 were analyzed. Thirty-seven patients had received prior therapy with one or a combination of the following agents: pentostatin, fludarabine, cladribine, chlorambucil, cyclophosphamide, doxorubicin, vincristine, prednisone, etoposide, or bleomycin, however, none had achieved CR. Patients received 3 or 10 mg initial test dose followed by gradual dose escalation of up to 30 mg three times per week until maximal response was achieved. The median dose received was 508 mg (range, 50-1325 mg) and the median duration of therapy was 35 days (range, 2-82 days). Thirty-eight patient outcomes were analyzed as one patient died prior to completion of treatment. The study results were: overall survival rate of 76%, CR 60%, partial remission (PR) 16%, and median DFI (disease-free interval ) of seven months (range 4-45 months). Factors associated with poor response were the presence of serous effusion, hepatic and CNS involvement, and very bulky lymphadenopathy. There were no statistically significant differences in response by age, sex, and presenting WBC count. CAMPATH-1H is considered to be the first-line treatment in T-PLL, however, it is not curative. Relapse is almost inevitable, all but two of the patients followed for over a year had relapsed [12].

Pentostatin: this is a purine analog that blocks adenosine deaminase (ADA), a key enzyme in the metabolism of the purines. When this enzyme is inhibited, adenosine and guanosine are accumulated and the proper degradation and salvage pathway is impaired leading to a decrease in DNA replication and cell growth. The pentostatin is added to the alemtuzumab therapy for patients who fail to attain complete remission [13]. In a phase II study that examined the efficacy and safety of the concurrent administration of alemtuzumab and pentostatin in patients with T-cell malignancies, 13 patients with T-PLL were included. Among the 13 patients with T-PLL, eight patients had prior treatment including single-agent pentostatin or alemtuzumab. The treatment protocols used for both drugs were: pentostatin at 4 mg/m2 intravenously (IV) weekly for four weeks then every two weeks until one of the following reached a CR or best response or 10 more doses; alemtuzumab initial dose was started one hour after the first dose of pentostatin and continued according to protocol (3 mg IV on day 1, 10 mg IV on day 2, 30 mg IV on day 3 and then 30 mg IV three times weekly until the achievement of CR, best response or a total of three months). The study result showed an overall response rate of 69% ( nine out of 13 patients), complete response 62% (eight out of 13 patients), and partial response 8% (one out of 13 patients). The median overall survival, progression-free survival, and event-free survival times in this study were 10.2 months, 7.8 months, and 20 months, respectively. As per this study, the response rates and duration of a combination of alemtuzumab and pentostatin was found to be superior to single-agent therapy [14].

Fludarabine, mitoxantrone, and cyclophosphamide (FMC): first-line chemotherapy in patients intolerant to alemtuzumab single agent or followed by alemtuzumab with bulky tumors, splenomegaly, and/or hepatic involvement. A phase II trial that assessed the response, survival, and toxicity of a sequential chemoimmunotherapy in T-cell PLL concluded that it is safe and effective. This therapy’s regimen included induction with fludarabine, mitoxantrone, and cyclophosphamide, for up to four cycles, followed by consolidation with alemtuzumab for up to 12 weeks. In intention to treat analysis overall response rate was 92% (12 complete remissions; 11 partial remissions) with median overall survival and progression-free survival 17.1 months and 11.9 months respectively [15].

Post-Remission Therapy 

Allogeneic or autologous hematopoietic stem cell transplantation (SCT) in eligible patients (30-50%): This is the standard of care in transplant-eligible patients after achieving complete response with first-line treatment (CR1). It has the potential for long-term disease control in this disease with an otherwise short relapse-free interval [16]. Retrospective studies have reported a wide range of survival differences with four-year progression-free survival (PFS) and OS been reported around 45% and 56% respectively with allogeneic SCT in this small study of 11 patients with mostly (9/11) in CR1 [17], whereas in other studies, it was reported slightly lower as three-year PFS and OS of 19-26% and 21-36% with 27-50% of the patients in CR1 [18]. Another study evaluating CIBMTR (Center for International Blood and Marrow Transplant Research) data has reported one-year PFS rates of 33% with allo-SCT [19]. The eligible patients of T-PLL for allo-SCT have been reported only in the range of 30-50% due to poor performance status [16]. A recent prospective observational study of 37 patients who received Alemtuzumab (95%) followed by allo-SCT, reported four-year non-relapse mortality (NRM of 32%), relapse rate (38%), PFS (30%), and OS (42%). Total body irradiation as a part of the conditioning regimen was the only statistically significant factor associated with lower relapse rates. Whereas the interval of more than 12 months between diagnosis and SCT was associated with lower NRM [20]. Delayed loss of chimerism without impact on initial engraftment has been reported possibly secondary to previous alemtuzumab use [17], which was successfully treated with donor lymphocyte infusions. 

Autologous SCT has also been attempted as a consolidation method with a reported median OS of 52 months in one of the retrospective studies in comparison to 33 months of median OS in the allo-SCT group and 20 months of median OS in the non-SCT group, although the difference was non-significant statistically [21].

## Review

Newer approaches of relapsed/refractory T-PLL disease

Alemtuzumab remains the gold standard therapy for the treatment of new, relapsed, and refractory disease. The first approach as of now consists of repeating single-agent alemtuzumab. Bendamustine, an alkylating agent, has shown to be effective as well when used as a single therapy in these cases. A multicenter retrospective study of 15 patients treated with bendamustine between 2009 and 2013 showed that bendamustine is an effective option for T-cell PLL refractory to alemtuzumab. In this retrospective study, 60% of the patients had prior treatment, including alemtuzumab. Patients included in the review were given bendamustine intravenously (70-120 mg/m2 /d on days one and two, every three weeks) for a total of six cycles. The overall response rate was 53.3 % with three patients (20%) achieving CR and five patients (33.3%) achieving a PR. The median PFS and OS were 5 and 8.7 months respectively. Moreover, the study reported PFS and OS were not correlated to alemtuzumab exposure [22].

The alemtuzumab-resistant relapse form of T-PLL is inevitable, and an approach to increase the sensitivity to alemtuzumab is reasonable. The combination of alemtuzumab and cladribine with or without epigenetic therapy (histone deacetylase [HDAC] inhibitors) can overcome alemtuzumab as demonstrated in a recent study. The rationale they used was based on other similar data and a phase II clinical trial that demonstrated the synergistic effects between cladribine, vorinostat, and monoclonal antibody rituximab in the aggressive B cell malignancy mantle cell lymphoma (MCL). Eight patients were treated with both cladribine and alemtuzumab in combination and seven achieved CR. Patient 1 had a relapse disease after alemtuzumab single-agent treatment. Cladribine was added and achieved CR for one year followed by relapse and re-achieved CR with cladribine and alemtuzumab. Patient 2 presented with alemtuzumab-resistant relapse but went into remission after the addition of cladribine and vorinostat. Although he relapsed several times, his disease remained susceptible to treatment with alemtuzumab, cladribine, and vorinostat. Patients 3, 4, 5, 6, and 8 were treated with combination cladribine and alemtuzumab with or without vorinostat as well. With the exception of patient 3, who achieved PR, these patients also achieved CRs and subsequent relapses remained susceptible to treatment. Major toxicities were hematologic and immune suppression [23].

Newer approaches in the treatment of relapsed/refractory T-PLL are based on recognizing the cytogenetic phenotype and targeting the identified defective genes involved in cell cycle regulation such as protooncogenes, tumor suppressor, and DNA repair genes. T-PLL exhibits mutations leading to the abnormal activation of TCL1 or MTCP1 protooncogenes, the JAK-STAT pathway, inactivation of the ATM gene, and other somatic mutations that are candidates for targeted therapies. The median survival of patients is less than two years and clinical trials are difficult to execute, hence the majority of researchers are using an ex vivo drug testing platform and subsequent in vivo trials in a small subset of patients [24].

BCL2 Inhibition

The B-cell lymphoma 2 (BCL2) family protein intervenes in the intrinsic apoptotic pathway of the mitochondria, a pathway required for normal embryonic development and prevention of cancer. Hence the inhibition of this protein will control cell growth by promoting cell death [25]. Venetoclax, a BCL2 inhibitor, was used ex vivo along with other drugs in 86 patients with refractory disease demonstrating the strongest T-PLL specific response. Ex vivo response to venetoclax, significantly correlated with the expression of the BCL2 protein. In the same study, similar results were obtained in vivo in two patients with late-stage refractory disease when used as a single agent. In patient 1, partial remission was achieved which can be translated as a decrease in splenomegaly and a good tolerance to the medication. The treatment had to be interrupted due to sepsis. In patient 2, the response was evidenced by the restitution of the splenomegaly and complete regression of lymphadenopathy. After some time, during treatment, the patient had a relapse which overall will account as partial remission only [26]. Similar to the approach that combines a purine analog with alemtuzumab, a case was reported implementing the use of a purine analog (pentostatin) with venetoclax, a novel combination used in a patient with refractory and relapse disease, which resulted in a durable complete remission [27]. Furthermore, a clinical trial in phase II is evaluating the efficacy of venetoclax plus Ibrutinib, the latter being a small molecule that binds permanently to the Bruton’s tyrosine kinase interfering with the B cell differentiation. This study includes all patients with active T-PLL and/or those that have been treated with alemtuzumab in the past [28].

JAK-STAT Pathway Inhibition

The Janus kinase/signal transducers and activators of transcription (JAK/STAT) signaling pathway mediate the cellular response to cytokines and growth factors. After the binding of interleukin-2 (IL2) to its correspondent surface receptor, the JAK-STAT pathway activation occurs leading to a cascade of phosphorylation. Activated STAT enters the nucleus and binds to specific enhancer sequences in target genes, thus regulating their transcription. This will result in the activation of the proliferation and function of T lymphocytes and natural killer cells [29]. Targeting the JAK-STAT pathway in the treatment of T-PLL is an important approach given that it constitutes more than 70% of the actionable mutations landscape in this disease [24]. The majority of these mutations are attributed first to STAT5B and second to JAK3. Alexandra Gomez-Arteaga et al. reported a case of refractory T-PLL with a JAK3 mutation treated with tofacitinib and ruxolitinib combined showing moderate activity [30]. In vitro selective inhibition of STAT5B [3] with pimozide showed specific and profound decreased cell proliferation in the cell lines expressing mutated IL2G-JAK-STAT (IL2RG, JAK1, JAK3, and STAT5B mutations) genes as opposed to other inhibitors of this axis such as tofacitinib, whose use didn’t result in selective tumor cells killing. A clinical trial in phase I intend to use itacitinib (a JAK1 inhibitor) combined with alemtuzumab in T-PLL patients. 

Oncogene Activation: TCL1 and MTCP1

The protein kinase B or AKT1 is involved in the PI3K/AKT/mTOR pathway, an intracellular signaling pathway directly related to cellular quiescence, proliferation, cancer, and longevity. AKT1 requires a cofactor named T-cell leukemia/lymphoma 1 (TCL1) which is expressed in normal immature B and T cells and is also associated with the MTCP1 gene [31]. Overexpression of the TCL1 gene in humans at chromosome 14q32.1 is considered as the major recurrent genomic abnormality in T-PLL associated with inversion [inv(14)(q11;q32)] and translocation [t(14;14)(q11;q32)]. The protein kinase B (AKT1) inhibitors (eg. MK 2206) cause disruption of the Akt transduction axis, associated with protooncogenes TCL1 or MTCP family, which in terms will inhibit the cell growth. Its efficacy has been tested in vitro using leukemic T cells. Furthermore, there are promising results from a phase II clinical trial in 59 patients with relapsed or refractory lymphoma that together with the in vitro results put MK-2206 as a potential therapeutic drug in T-PLL [1].

Histone Deacetylase (HDAC) Inhibition

Histone deacetylases (HDACs) are enzymes that remove acetyl groups from histones. Apart from regulating histone modification, HDACs also regulate the post-translational acetylation status of many non-histone proteins. Deacetylation of non-histone proteins by HDACs results in degradation via the ubiquitin-proteasome pathway. The acetylation status of RUNX3, a tumor suppressor and transcription factor, is important for its stability and transcriptional activity, which is enhanced by HDAC inhibitors [32]. Vorinostat, belinostat, and romidepsin are FDA approved for the treatment of a variety of T-cell lymphomas. The combination with cladribine increased the sensitivity to alemtuzumab and decreased prior treatment resistance in all treated patients where seven out of eight achieved CR. Other approaches focus on the combination of HDAC inhibition with DNA-damage induction, e.g., panobinostat combined with bendamustine or the fusion molecule tinostamustine (vorinostat and bendamustine) that already has FDA fast-track approval [16].

PARP Inhibitors

Poly(ADP-ribose) polymerase (PARP) is a nuclear enzyme that plays a critical role in the repair of any single-strand DNA damage via the base excision repair pathway. PARP inhibitors such as olaparib, have substantial single-agent antitumor activity by inducing synthetic lethality. This concept requires simultaneous inactivation of two genes to cause cell death. In T-PLL, tumor cells have a dysfunctional ATM which works as a DNA repair gene sensitizing tumor cells to DNA damage agents. The inability to repair the DNA along with the dysfunctional polymerase activity will lead to cell death. Olaparib has been used on the basis of this same concept of synthetic lethality in patients with breast or ovarian cancer associated with deleterious BRCA1 or BRCA2 mutation where the use of PARP inhibitors was evidenced to be effective [1].

CAR-T Therapy

The Chimeric Antigen Receptor T-cells (CAR-T) are T-cells that have been modified in the laboratory to generate a surface receptor that is able to bind a specific antigen. This therapy is raising interest in the field of T-cell malignancies. Normal T-cells express T-cell receptor Beta chain constant region 1 or 2 (TRBC1/TRBC2) with a near 50:50 distribution among the healthy T-cells while malignant cells are clonally TRBC1 positive. CAR-T-cells targeting TRBC1 of malignant clones killed only T-PLL cells while sparing healthy T-cells in vitro [16]. This approach offers the potential to spare an adequate number of normal T-cells to maintain polyclonal T-cell immunity.

Future prospects of targeted therapies in T-PLL

Targeted epigenetic therapies (such as EZH2 inhibitors), tumor suppressor (CHEK2, FBXW10, and SAMHD1) enhancers, P53 de-repressors, and many other rationale-based techniques are currently being studied with potential for future treatment in many hematologic malignancies including T-PLL [16, 33-37].

## Conclusions

T-PLL is a rare hematologic neoplasm that encounters one of the most common challenges of many rare diseases: the lack of sufficient data for evidence-based practice of medicine and innovative means of management. However, it is imperative to continue the study and the expansion of the knowledge in the treatment of those pathologies that affect not a percentage in the statistics but in general the human beings. The advent of genetic therapies has promised to lead the future of pharmacologic therapies for these otherwise not so popular diseases that remain out of the focus of the researchers.
